# A colourful duplication

**DOI:** 10.7554/eLife.92763

**Published:** 2023-11-02

**Authors:** Violaine Llaurens

**Affiliations:** 1 Centre national de la recherche scientifique (CNRS) Paris France; 2 Muséum national d'Histoire naturelle Paris France

**Keywords:** arctia plantaginis, colour, polymorphism, wing patterns, evolution, genetics, Other

## Abstract

A genetic duplication event during evolution allowed male wood tiger moths to have either yellow or white patterns on their wings.

**Related research article** Brien MN, Orteu A, Yen EC, Galarza JA, Kirvesoja J, Pakkanen H, Wakamatsu K, Jiggins CD, Mappes J. 2023. Colour polymorphism associated with a gene duplication in male wood tiger moths. *eLife* **12**:e80116. doi: 10.7554/eLife.80116.

The beautiful patterns found on the wings of moths and butterflies can provide important insights into adaptive evolution (Orteu and Jiggins, 2020). Natural selection exerted by predators, and sexual selection exerted by females, have led to the emergence of an incredible diversity of new colours and patterns (Briolat et al., 2019; Robertson and Monteiro, 2005). While some of the genes involved in this diversification have recently been identified (Van’t Hof et al., 2016; Livraghi et al., 2021), it is still unclear whether the emergence of new colours and patterns is generally driven by similar sets of genes or by the evolution of new ones.

Within some species, individuals can have strikingly different colour patterns on their wings. For instance, male wood tiger moths can have either yellow or white hindwings, with both types of males usually occurring within the same geographical location (Figure 1A). Understanding the genetic mechanisms that allow different coloured individuals to co-exist within a population – a phenomenon called colour polymorphism – can help identify how new traits emerged over the course of evolution (Llaurens et al., 2017). Now, in eLife, Chris Jiggins, Johanna Mappes and co-workers – including Melanie Brien (University of Helsinki) and Anna Orteu (University of Cambridge) as joint first authors – report which genes determine whether a male wood tiger moth will develop white or yellow hindwings (Brien et al., 2023).

**Figure 1.**
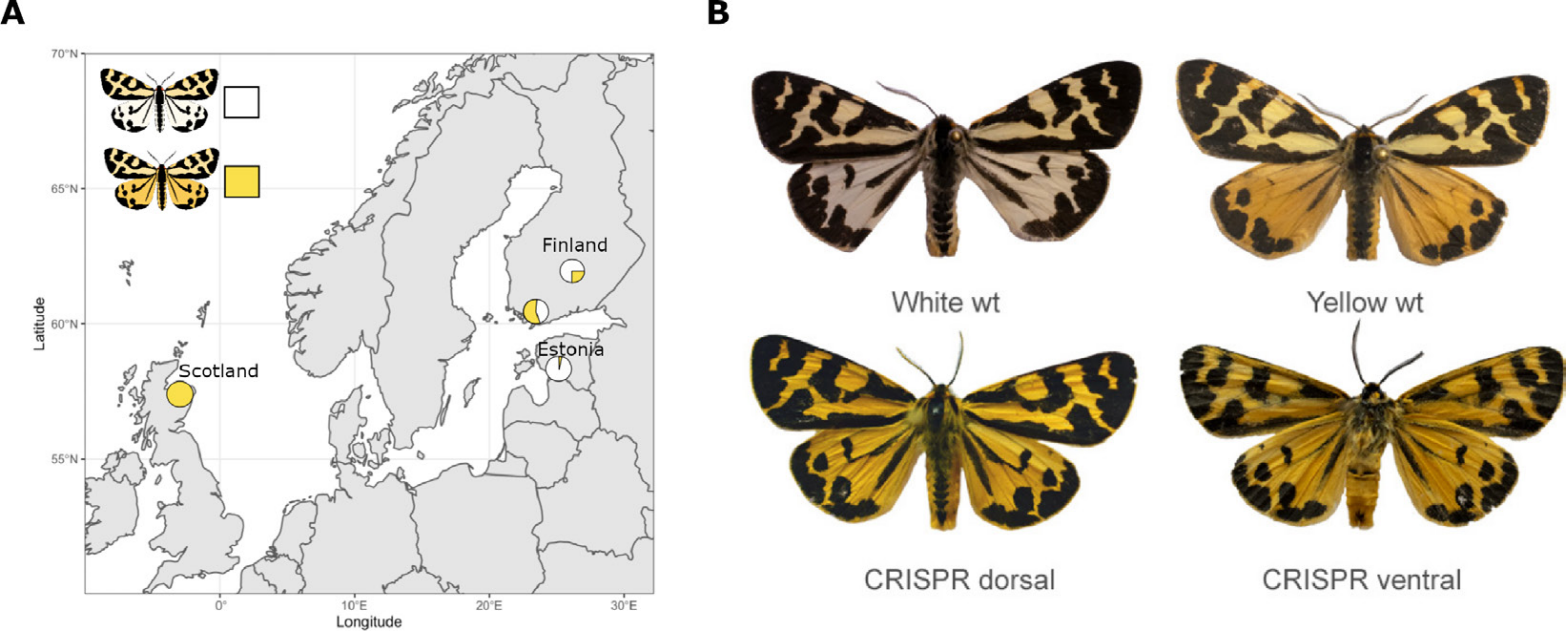
Finding the gene responsible for colour polymorphism in wood tiger moths. (**A**) The proportion of male wood tiger moths that have yellow or white hindwings varies between geographical locations. For instance, 40–70% of males living in Finland and 97% of males living in Estonia have white hindwings, whereas all male moths in Scotland have yellow hindwings. (**B**) To investigate if the gene *valkea* was responsible for this colour polymorphism, Brien et al. used the genetic tool CRISPR-Cas9 to modify its sequence in wild-type (wt) moths with white hindwings (top left). This caused the mutant males to display yellow instead of white on the back (bottom left) and front (bottom right) of their hindwings, similarly to wild-type yellow moths (top right). The forewings of the genetically modified moths were also more yellow than wild-type white males, which is likely due to the CRISPR-Cas9 modification also introducing a mutation in to the full-length copy of the *yellow-e* gene.

Image credit: Adapted from Figure 1 and Figure 4 of Brien et al., 2023.

Like detectives working on a complex case, the team patiently gathered several lines of evidence to find the genetic variations responsible for this colour polymorphism. By crossing yellow females with white males, they were able to identify a region of the genome that is associated with colour differences in male offspring. This region contains 21 genes, including four from the *yellow* gene family, and is a different size in yellow and white males. In white males, this part of the genome consists of a large duplicated area which contains both a full-length copy and truncated copy of the *yellow-e* gene. Brien et al. hypothesized that the truncated gene is responsible for the white phenotype in the wood tiger moth, and named the suspected gene *valkea*, the Finnish word for white.

Next, Brien et al. studied the genes expressed in the wings of caterpillars and pupae before they grow into adult wood tiger moths. Several genes were found to be differentially expressed in the white and yellow moths: as expected, *valkea* was only turned on in white males. Interestingly, the full-length copy of the *yellow-e* gene was also overexpressed in the wings of the white males during this phase of development.

To confirm that *valkea* controls wing colour, Brien et al. introduced a ‘guide’ that allowed the gene editing tool CRISPR-Cas9 to specifically modify the DNA sequence of the *valkea* gene. More than 1000 eggs from white moths were injected with the *valkea*-specific guide, but only six individuals reached adulthood, with four out of five male adults developing partially yellow hindwings (Figure 1B). Unfortunately, the guide also targeted the full-length copy of the *yellow-e* gene in addition to *valkea*, making it difficult to determine the respective role each of these genes play in colour polymorphism.

The findings of Brien et al. suggest that the hindwing colour of male wood tiger moths is determined by genes from the *yellow* family, which are known to regulate wing colour in other insects (Wittkopp et al., 2002). This highlights how the diverse range of colour patterns seen in winged insects are determined by only a small number of genes (Zhang et al., 2017). The results also confirm the important role of gene duplication in driving the evolution of new traits (Martin and Reed, 2010).

The re-use and duplication of the *yellow-e* gene can be interpreted as evolutionary tinkering (Jacob, 1977). The tinkering of such a small set of genes demonstrates how a few genetic variations can generate such a striking diversity of phenotypes. Nevertheless, the small set of genetic variations that can be targeted by selection also exert strong constraints on adaptive evolution, potentially limiting the range of colours and patterns that can emerge over the course of evolution.

Distinguishing the respective roles of similar genes is challenging. The work of Brien et al. is a promising step in investigating the exact changes within and among gene copies that led to individual members of a species developing one trait or another. This type of work on polymorphism could be combined with research investigating how gene duplications impact the evolution of phenotypes between different species. The results of these studies could highlight the different evolutionary paths responsible for the diverse colours and patterns seen on the wings of moths and butterflies.
